# PTPRT Could Be a Treatment Predictive and Prognostic Biomarker for Breast Cancer

**DOI:** 10.1155/2021/3301402

**Published:** 2021-08-10

**Authors:** Lun Li, Feng Xu, Pingfang Xie, Liqin Yuan, Meirong Zhou

**Affiliations:** Department of General Surgery, Xiangya Second Hospital, Central South University, No. 139 Middle People Road, Changsha, Hunan 410011, China

## Abstract

The role of PTPRT in breast cancer was not comprehensively explored and well analyzed. Our study comprehensively searched available databases to analyze the clinical role of PTPRT in breast cancer. We found PTPRT was an antioncogene and could be used to distinguish different stages, age groups, molecular types, and grades for breast cancer. PTPRT might be primary resistance biomarkers for taxane, anthracycline, and ixabepilone but not be acquired resistance biomarkers. Higher PTPRT expression levels were associated with longer overall survival and recurrence-free survival. PTPRT was negatively associated with Ki67 and CDK4/6 but positively associated with BCL-2. PTPRT might be associated with cell cycle and microtubule, and tumor infiltration in B cell and macrophage cell. PTPRT could predict chemotherapy effectiveness and prognosis for breast cancer patients. PTPRT might inhibit tumor growth via disrupting the microtubule dynamics and cell cycle in breast cancer.

## 1. Introduction

PTPRT belongs to the type IIB RPTP subfamily, which consisted of an extracellular domain (a meprin/A5/PTP *μ* domain, an Ig domain, and four fibronectin type III repeats), a transmembrane domain, a juxtamembrane region, and two phosphatase domains (D1 and D2) [1]. PTPRT plays in suppressing tumor growth and cell adhesion in various cancers, including colorectal cancer [2], hepatocellular carcinoma [3], prostate cancer [4], lung squamous cell carcinoma [5], esophageal squamous cell carcinoma [6], and glioma [7]. Previous review showed PTPRT as a tumor suppressor might be involved in cell cycle and cell adhesion [1]. Five missense mutations in the most commonly altered PTPRT were found to reduce phosphatase activity, and expression of wild-type but not a mutant PTPRT in human cancer cells inhibited cell growth [8]. Zhang et al. showed deletion of the fibronectin type III repeats (FNIII) of PTPRT result in defective cell-cell aggregation, which suggest the inactivation of PTPRT might lead to cancer progression by disrupting cell-cell adhesion [9]. Available studies about the PTPRT were limited, and about 66 studies were found in Pubmed. Few studies were conducted about the PTPRT signaling pathway. Zhang et al. identified signal transducer and activator of transcription 3 (STAT3) as a substrate of PTPRT. They showed PTPRT specifically dephosphorylated STAT3 at a tyrosine at amino acid Y705 and overexpression of normal PTPRT in colorectal cancer cells reduced the expression of STAT3 target genes [10]. Other studies identified miR-532-3p [3], miR-218 [6], miR-215 [11], and miR-888 [12] might regulate and mediate the expression of PTPRT. Schettini et al. used a novel methodology to detect surface antigen to develop ADC and CAR-T against breast cancer already and identified PTPRT as a novel potential target for molecular Luminal A or immunohistochemical HR+/HER2-negative BC [13]. In order to analyze the clinical role of PTPRT in breast cancer, we comprehensively searched available databases to summarize the treatment predictive and prognostic values of PTPRT.

## 2. Materials and Methods

No Institutional Review Board (IRB) approval was needed for this study. Available databases based on TCGA and GEO data were searched using PTPRT and breast cancer. Three databases based on TCGA data including GEPIA (http://gepia.cancer-pku.cn/), UALCAN (http://ualcan.path.uab.edu/analysis.html), and Linkedomics (http://www.linkedomics.org/login.php) were used to analyze the differences of PTPRT expression in different age groups, stages, and molecular types, as well as the prognostic value of PTPRT in breast cancer. Three databases based on GEO data including bc-GenExMiner v4.3 (http://bcgenex.centregauducheau.fr/BC-GEM/GEM-requete.php), ROCPLOT (http://www.rocplot.org/user/login), and KMPLOT (http://kmplot.com/) were used to analyze the differences of PTPRT expression in different age groups, stages, and molecular types, as well as the predictive values of different drugs and the prognostic value of PTPRT in breast cancer.

Three databases based on TCGA data about DNA methylation including UALCAN (http://ualcan.path.uab.edu/analysis.html), Wanderer (http://maplab.imppc.org/wanderer/), and Methsurv (https://biit.cs.ut.ee/methsurv/) were used to retrieve the CpG sites of PTPRT and their prognostic roles. GEO datasets (https://www.ncbi.nlm.nih.gov/gds/) were also searched to obtain the PTPRT expression in different acquired resistance cell lines and the relationships between PTPRT and ESR1, PGR, ERBB2, KI67. Other genes that might be associated with PTPRT were explored using Cytoscape based on available public databases.

The most correlated coexpressed genes were retrieved based on linkedomics database (*r* > 0.4 or *r* < −0.4, *p* < 0.05). These genes were then submitted to Gprofiler (https://biit.cs.ut.ee/gprofiler/gost) for GO enrichment analysis and Kobas (http://kobas.cbi.pku.edu.cn/anno_iden.php) for KEGG analysis. TIMER (https://cistrome.shinyapps.io/timer/) is a comprehensive resource for the systematical analysis of immune infiltrates across diverse cancer types.

## 3. Results

### 3.1. The Expression of PTPRT in Breast Cancer

**Figure 1:**
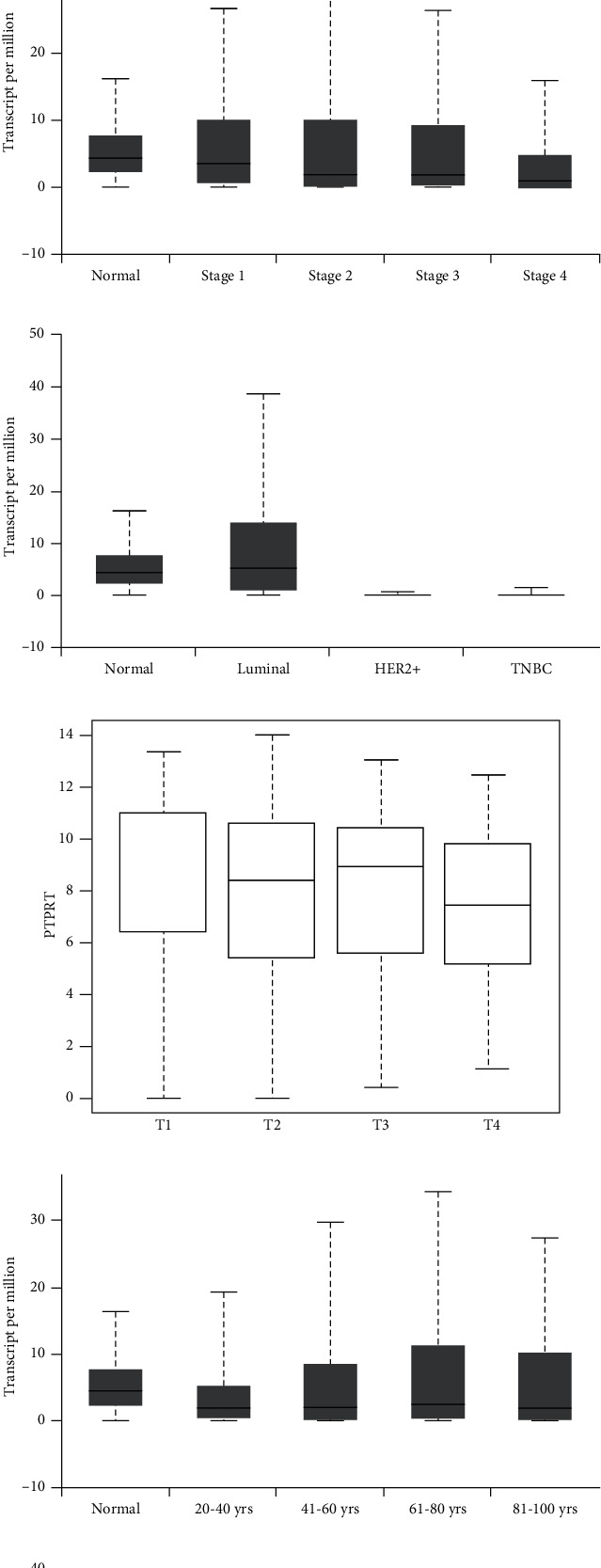
The expression of PTPRT in breast cancer in TCGA. (a) The expression of PTPRT between tumor and normal breast cancer tissue. (b) The expression of PTPRT in breast cancer across different molecular types. (c) The expression of PTPRT in breast cancer across different age groups. (d) The expression of PTPRT in breast cancer across different stages. (e) The expression of PTPRT in breast cancer across different tumor sizes. (f) The expression of PTPRT in breast cancer across different node statuses.

Using TCGA data, the expression level of PTPRT in breast cancer tissue is lower than that in adjacent normal breast tissue (median 2.24 vs. 4.41 TPM (transcript per million), *p* < 0.001). The expression level of PTPRT in stage 1 to 4 breast cancer tissues was lower than that in adjacent normal breast tissue (stage 1 vs. stage 2 vs. stage 3 vs. stage 4: 3.68 vs. 2.01 vs. 1.92 vs. 0.83). The expression level of PTPRT decreased from stage 1 to stage 4, and there were statistical significances between stage 1 and 4, stage 2 and 4 (*p* < 0.05). Interestingly, the expression level of PTPRT increased with age from 1.80 TPM in patients aged 20 to 40 years to 2.41 TPM in patients aged 61 to 80 years. Luminal A/B breast cancer patients have higher PTPRT expression level than that in adjacent normal breast cancer tissues (5.21 vs. 4.41, *p* < 0.001), while HER2+ (median 0.16 vs. 4.41, *p* < 0.001) and TNBC (median 0.09 vs. 4.41, *p* < 0.001) patients have lower PTPRT expression than that in adjacent normal breast cancer tissues (Figure 1).

**Figure 2:**
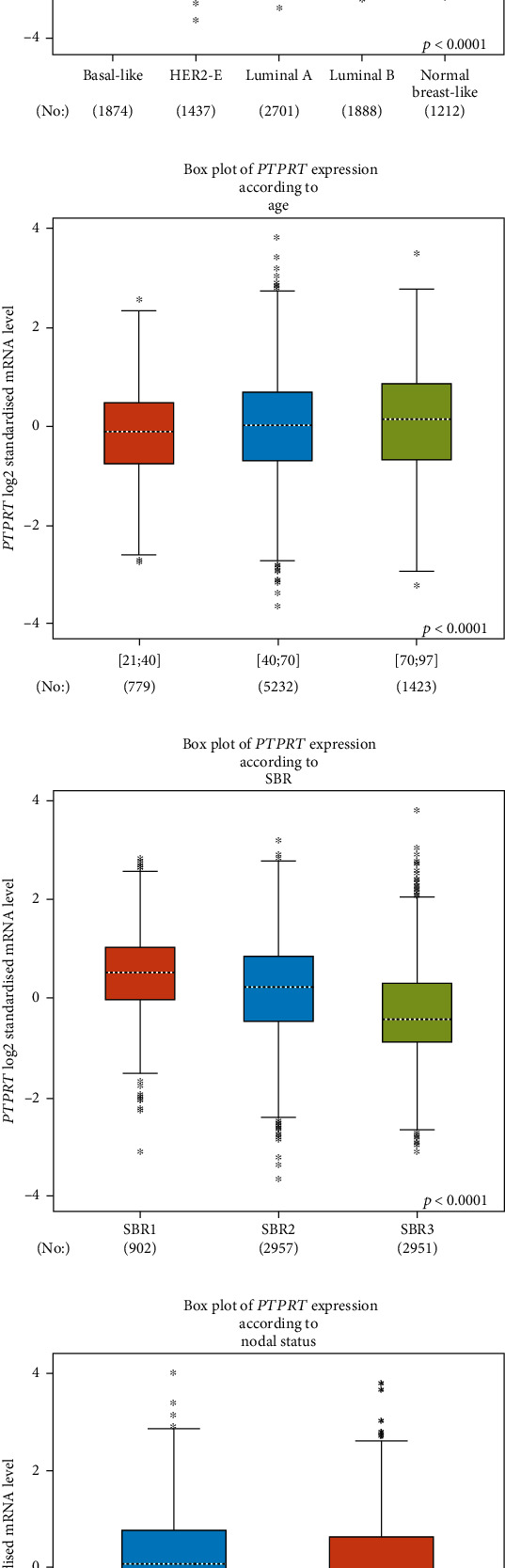
The expression of PTPRT in breast cancer based on GEO data. (a) The expression of PTPRT in breast cancer across different molecular types. (b) The expression of PTPRT in breast cancer across different age groups. (c) The expression of PTPRT in breast cancer across different SBR groups. (d) The expression of PTPRT in breast cancer between different node statuses.

Based on TCGA data, PTPRT was higher in pT1 breast cancer patients (RNA-Seq by Expectation-Maximization (RSEM), log2, median 9.46, IQR 6.42-11.02) than those in pT2 (median 8.41, IQR 5.40-10.60) and pT4 (median 7.45, IQR 5.18-9.85), and similar to that in pT3 breast cancer patients (median 8.92, IQR 5.58-10.46). However, there were statistical significances in the PTPRT expression levels between pathologic N0 (median 2.30, IQR 0.27 9.03), N1(median 2.43, IQR 0.36 11.31), N2 (median 1.80, IQR 0.34 8.98), and normal (median 4.41, IQR 2.38 7.70); no significance was found between N3 (median 2.42, IQR 0.23 7.76) and normal (Figure 1). Based on GEO data, the expression level of PTPRT decreased with the increase of Scarff-Bloom-Richardson (SBR) grade (SBR1 > SBR2 > SBR3, *p* < 0.001, 6810 patients). Those patients with lymph node metastases were of lower PTPRT levels (*p* < 0.0001, 7474 patients). The PTPRT expression levels in patients with different ages were similar to that in TCGA databases. The older the patients, the higher the PTPRT expressions (7434 patients, 70 − 97 > 40 − 70 > 21 − 40, *p* < 0.05). Luminal A breast cancer patients were of the highest level of PTPRT, which was higher than that in normal-like breast cancer (*p* < 0.05). HER2+ and basal-like breast cancer were of lower PTPRT level than that in normal-like breast cancer, and basal-like breast cancer was of the lowest PTPRT expression level. There was no statistical significance between luminal B and normal-like breast cancer (Figure 2).

### 3.2. The Promoter Methylation Level of PTPRT in Breast Cancer

**Figure 3:**
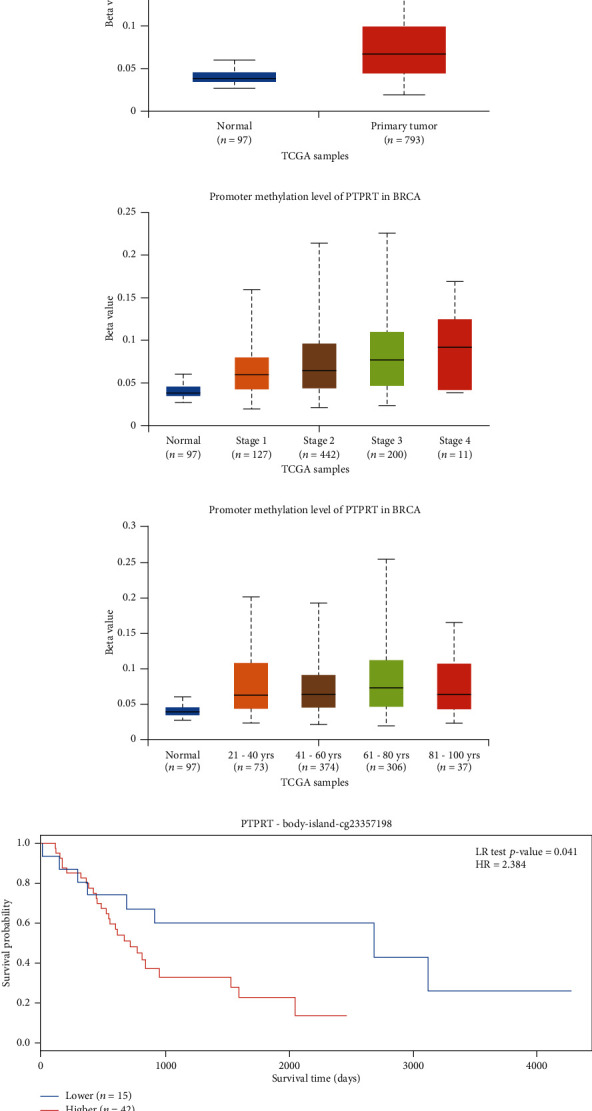
The methylation expression of PTPRT in breast cancer based on TCGA data. (a) The methylation expression of PTPRT between tumor and normal breast cancer tissue. (b) The methylation expression of PTPRT in breast cancer across different stage groups. (c) The methylation expression of PTPRT in breast cancer across different age groups. (d) The relationship of PTPRT methylation levels and overall survival (cg23357198).

Based on TCGA data, the promoter methylation level of PTPRT was higher in breast cancer tissues than those in adjacent normal breast tissue (0.067 vs. 0.039, *p* = 1.62*E* − 12). The promoter methylation level of PTPRT increase from stage 1 to stage 4 (0.06 vs. 0.07 vs. 0.08 vs. 0.09); however, there was a statistical difference between stage 1 and 2 (*p* = 0.01). Also, this increased with the age (21-40 vs. 41-60 vs. 61-80: 0.06 vs. 0.06 vs. 0.07); however, statistical differences were only found between patients age 21 and 40 years old with 41-60, 61-80, or 81-100 years old. Only CpG site cg23357198 could predict survival (high vs. low: HR = 2.38, *p* = 0.04) (Figure 3).

### 3.3. The Predictive Values of PTPRT in Breast Cancer Treatment

Based on rocplot.plot, among those patients who received neoadjuvant taxane (AUC = 0.59, *p* = 3.1*e* − 7), anthracycline (AUC = 0.60, *p* = 1.1*e* − 11), ixabepilone (AUC = 0.61, *p* = 0.04), and FAC (AUC = 0.6, *p* = 6.6*e* − 3), the PTPRT expression level was higher in no-responders than those in responders. PTPRT might not predict the effectiveness of aromatase inhibitor (AUC = 0.54, *p* = 0.33), trastuzumab (AUC = 0.56, *p* = 0.09), lapatinib (AUC = 0.59, *p* = 0.13), FEC (AUC = 0.51, *p* = 0.44), and CMF (AUC = 0.52, *p* = 0.37).

**Figure 4:**
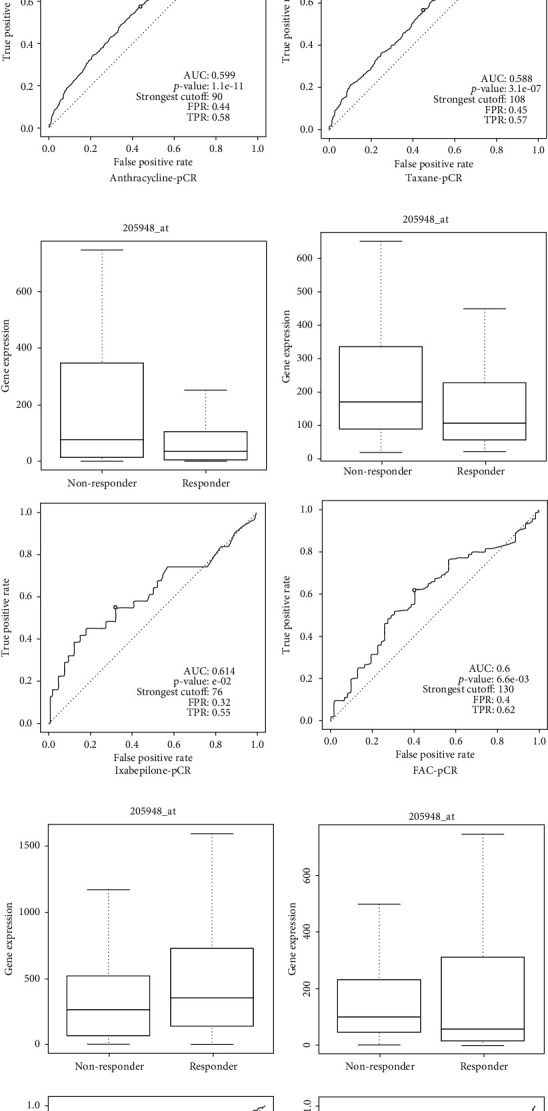
The predictive values of PTPRT in breast cancer for different drugs. (a) The predictive values of PTPRT for anthracycline response in the neoadjuvant setting. (b) The predictive values of PTPRT for taxane response in the neoadjuvant setting. (c) The predictive values of PTPRT for ixabepilone response in the neoadjuvant setting. (d) The predictive values of PTPRT for FAC response in the neoadjuvant setting. (e) The predictive values of PTPRT in RFS for those who received tamoxifen in the adjuvant setting. (f) The predictive values of PTPRT in RFS for those who received anthracycline in the adjuvant setting.

Among those patients who received adjuvant CMF (AUC = 0.6, *p* = 0.09), FAC (AUC = 0.53, *p* = 0.4), aromatase inhibitor (AUC = 0.61, *p* = 0.21), trastuzumab (AUC = 0.50, *p* = 0.48), taxane (AUC = 0.51, *p* = 0.14), and FEC (AUC = 0.56, *p* = 0.33), PTPRT might not predict the recurrence-free survival of the breast cancer patients. A high level of PTPRT might be associated with more survivors among those who received adjuvant tamoxifen (AUC = 0.59, *p* = 7.1*e* − 4), but fewer survivors in those who received anthracycline (AUC = 0.56, *p* = 2.9*e* − 2) (Figure 4).

### 3.4. The Prognostic Values of PTPRT in Breast Cancer

KM plot showed that high PTPRT expression levels were associated with longer survivals in terms of overall survival (HR 0.6, 95% CI 0.48 0.75, *p* = 5.1*E* − 6) and recurrence-free survival (HR 0.6, 95% CI 0.54 0.68, *p* < 1*E* − 16). This was consistent with the prognostic analysis results from TCGA and Breast Cancer Gene-Expression Miner v4.3 data-sets, which also confirmed the survival benefits of high PTPRT. The prognostic values of PTPRT were consistent across different molecular types of breast cancer.

### 3.5. The Expression of PTPRT between Acquired Drug-Resistant and Parental Cell Lines

PTPRT might not be acquired resistance biomarkers for tamoxifen (MCF, GSE26459, logFC 0.57, *p* = 0.09; GSE67916, logFC = −0.20, *p* = 0.13), epirubicin (MCF7, GSE54326, logFC = −0.64, *p* = 0.25; SKBR3, GSE54326, logFC = 1.27, *p* = 0.36; MDA-MB-231, GSE54326, logFC = 0.31, *p* = 0.65), trastuzumab (BT474, GSE15043, logFC = -0.01, p = 0.92; BT474, GSE119397, logFC = 0.007, *p* = 0.95), T-DM1 (BT474, GSE100192, logFC = −0.002, *p* = 0.98), lapatinib (BT474, GSE16179, logFC = −0.496, *p* = 0.70; SKBR3, GSE38376, logFC = 0.18, *p* = 0.14; SKBR3, GSE52707, logFC = 0.26, *p* = 0.03; BT474, GSE84896, logFC = 0.29, *p* = 0.001), paclitaxel (MDA-MB-231, GSE90564, logFC = −0.42, *p* = 0.07; MDA-MB-231, GSE12791, logFC = 0.87, *p* = 0.29) and BMS-554417 (MCF, GSE18912, logFC = −0.024, *p* = 0.79). However, PTPRT might be an acquired resistance biomarker for doxorubicin among MCF7 cell lines (MCF7, GSE76540, logFC = 1.12, *p* = 0.03).

### 3.6. The Relationship between PTPRT and ER, PR, HER2, Ki67

**Figure 5:**
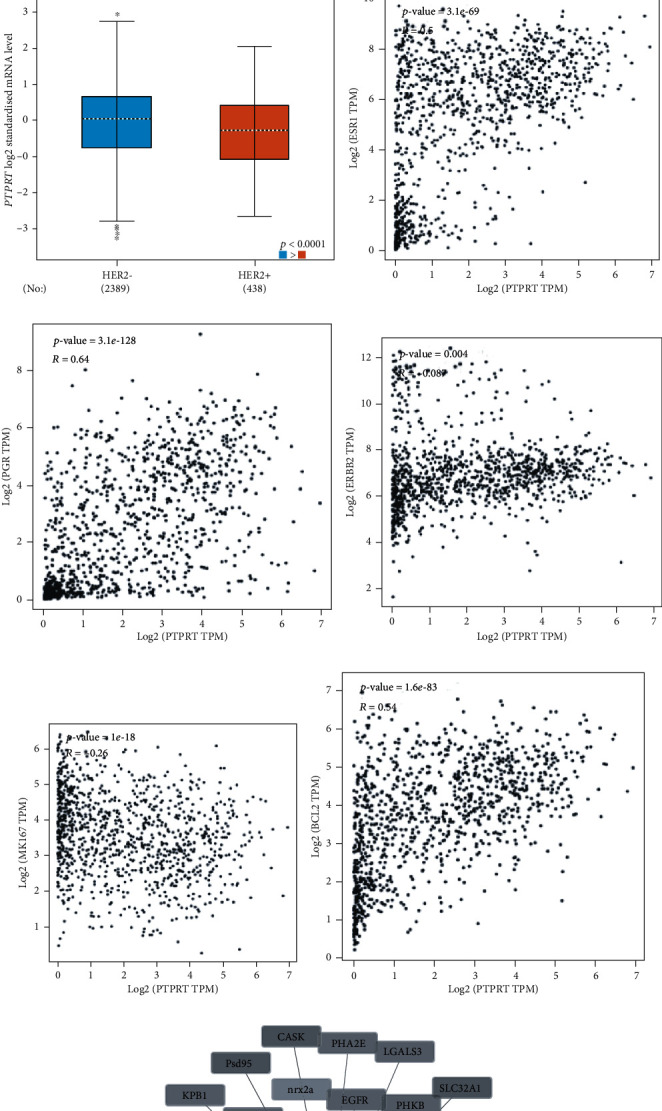
The relationship between PTPRT and ER, PR, HER2, Ki67, and BCL-2. (a) The expression of PTPRT between ER+ and ER- breast cancer. (b) The expression of PTPRT between PR+ and PR- breast cancer. (c) The expression of PTPRT between HER2+ and HER2- breast cancer. (d) The relationship between PTPRT and ER. (e) The relationship between PTPRT and PR. (f) The relationship between PTPRT and HER2. (g) The relationship between PTPRT and Ki67. (h) The relationship between PTPRT and BCL-2. (i) The genes that might be associated with PTPRT.

According to bc-GenExMiner v4.3, estrogen receptor or progesterone receptor-positive (IHC) breast cancer patients were with higher PTPRT levels, while HER2+ (IHC) breast cancer patients were with lower PTPRT level. According to TCGA data, PTPRT was positively associated with ESR1 (*R* = 0.5, *p* = 3.9*e* − 69), PGR (*R* = 0.64, *p* = 3.1*e* − 128); however, PTPRT was negatively associated with ERBB2 (*R* = −0.09, *p* = 0.004), KI67 (*R* = −0.26, *p* = 1*e* − 18). Meanwhile, PTPRT was positively associated with BCL-2 (*R* = 0.54, *p* = 1.6*E* − 83). Based on GEO datasets, estrogen receptor alpha knockdown (GSE37473, logFC = 0.06, *p* = 0.61) and HER2 siRNA (GSE71347, logFC = 0.22, *p* = 0.09) did not influence the expression of PTPRT (Figure 5).

### 3.7. PTPRT Might Inhibit Tumor Growth via Disrupting the Microtubule Dynamics and Cell Cycle

**Figure 6:**
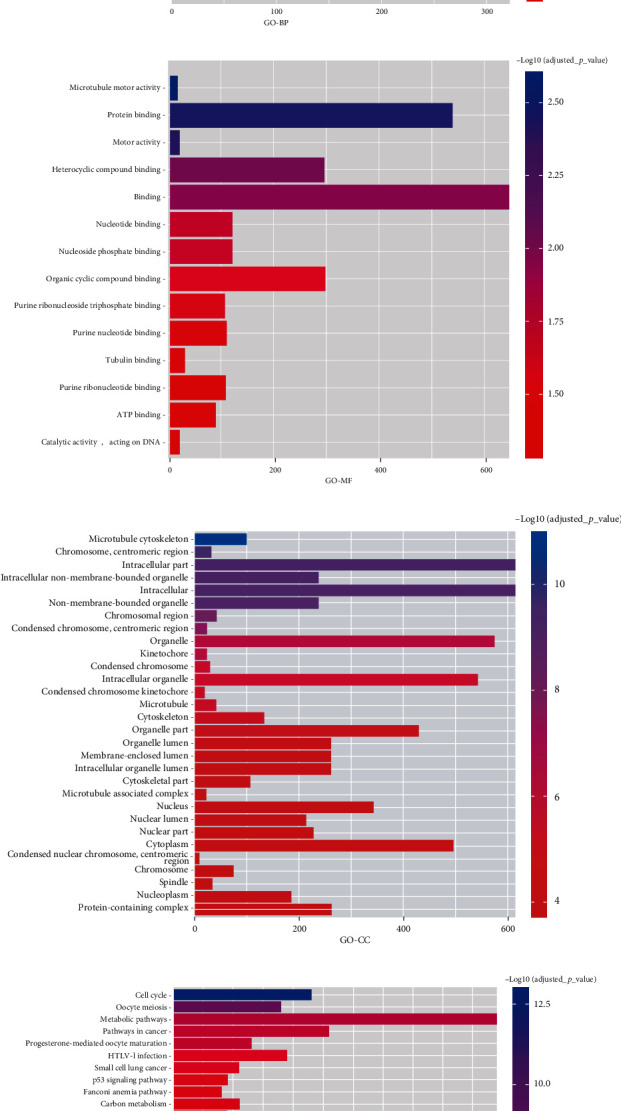
The enrichment analysis for PTPRT in breast cancer. (a) The biological process. (b) The molecular function. (c) The cellular component. (d) The KEGG pathway.

According to the KEGG and GO enrichment analysis of the genes that were associated with PTPRT (*r* > 0.4 or *r* < −0.4, *p* < 0.05), we found PTPRT might be associated with cell cycle and microtubule-based process. For biological process, PTPRT might be associated with the mitotic cell cycle, microtubule-based process, mitotic cell cycle process, cell cycle, cell division, microtubule cytoskeleton organization, and cell cycle process. For cellular component, PTPRT was associated with the microtubule cytoskeleton, while for molecular function, PTPRT was associated with microtubule motor activity, protein binding, and motor activity (Figure 6).

**Figure 7:**
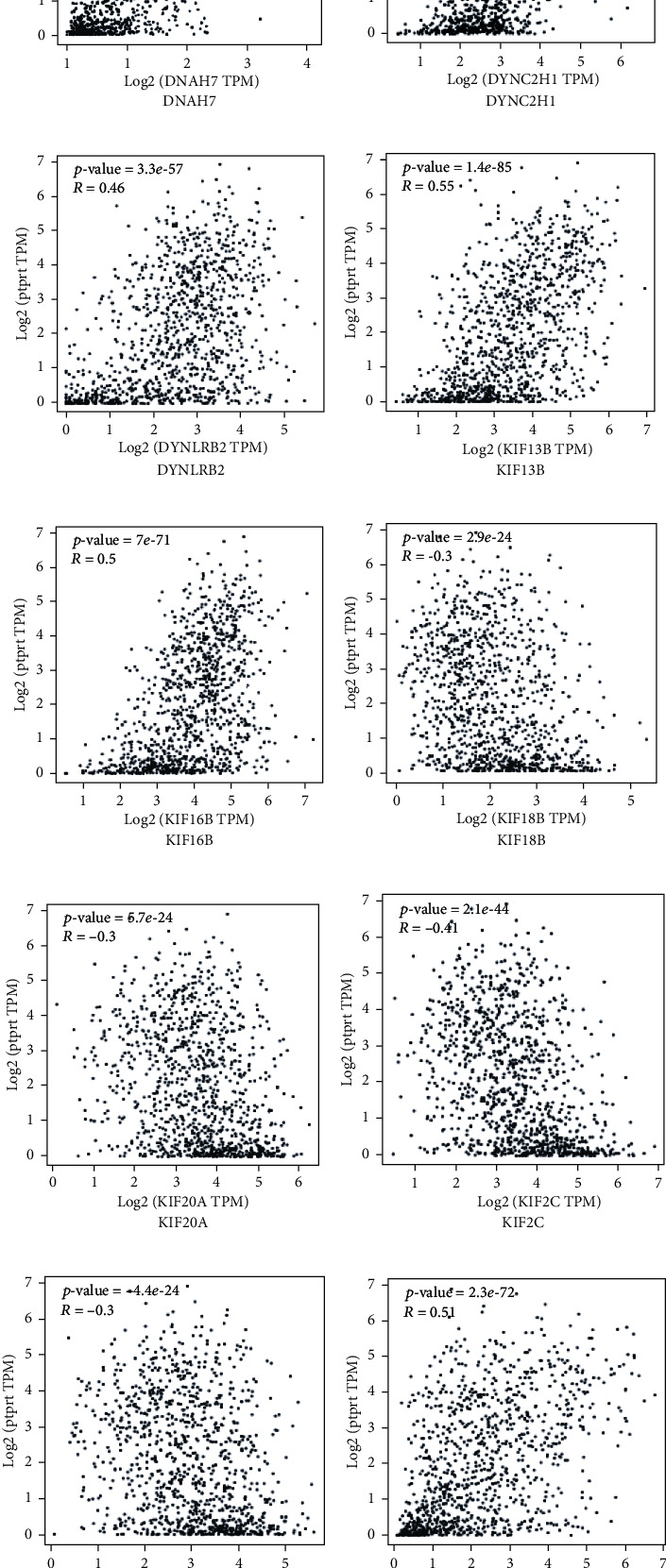
The relationship between PTPRT and microtubule-related genes. (a) The relationship between PTPRT and BBS4. (b) The relationship between PTPRT and DNAH5. (c) The relationship between PTPRT and DNAH7. (d) The relationship between PTPRT and DYNC2H1. (e) The relationship between PTPRT and DYNLRB2. (f) The relationship between PTPRT and KIF13B. (g) The relationship between PTPRT and KIF16B. (h) The relationship between PTPRT and KIF18B. (i) The relationship between PTPRT and KIF20A. (j) The relationship between PTPRT and KIF2C. (k) The relationship between PTPRT and KIF4A. (l) The relationship between PTPRT and KIF5C. (m) The relationship between PTPRT and KIFC1. (n) The relationship between PTPRT and WDR78.

Among the genes in microtubule motor activity (biological process, GO:0007017, microtubule-based process; molecular function, GO:0003777, microtubule motor activity; cellular component, GO:0015630, microtubule cytoskeleton), 14 genes (BBS4, DNAH5, DNAH7, DYNC2H1, DYNLRB2, KIF13B, KIF16B, KIF18B, KIF20A, KIF2C, KIF4A, KIF5C, KIFC1, WDR78) were in all these three categories. All these genes were significantly associated with PTPRT. Among them, BBS4 (*R* = 0.54, *p* < 0.001), DNAH5 (*R* = 0.45, *p* < 0.001), DNAH7 (*R* = 0.54, *p* < 0.001), DYNC2H1 (*R* = 0.55, *p* < 0.001), DYNLRB2 (*R* = 0.46, *p* < 0.001), KIF13B (*R* = 0.55, *p* < 0.001), KIF16B (*R* = 0.5, *p* < 0.001), KIF5C (*R* = 0.5, *p* < 0.001), and WDR78 (*R* = 0.44, *p* < 0.001) were positively associated with PTPRT, while KIF18B (*R* = −0.3, *p* < 0.001), KIF20A (*R* = −0.3, *p* < 0.001), KIF2C (*R* = −0.41, *p* < 0.001), KIF4A (*R* = −0.3, *p* < 0.001), and KIFC1 (*R* = −0.37, *p* = *p* < 0.001) were negatively associated with PTPRT (Figure 7).

Further, we analyzed the relationships between PTPRT and CDK4/6, and PTPRT was negatively associated with CDK4 (*r* = −0.34, *p* < 0.001), CDK6 (*r* = −0.25, *p* < 0.001), and MYC (*r* = −0.16, *p* < 0.001).

### 3.8. PTPRT in Tumor-Infiltrating Immune Cells

**Figure 8:**
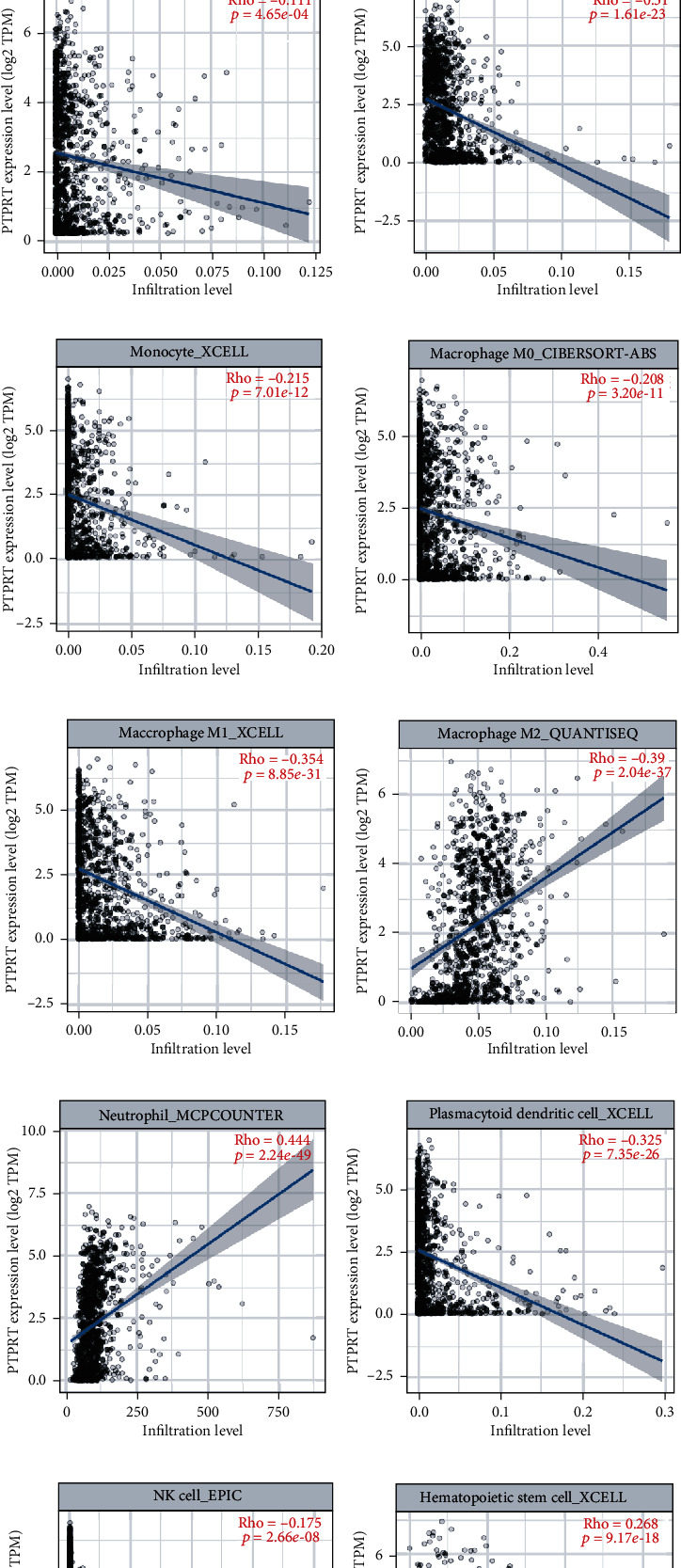
The relationship between PTPRT and tumor infiltration. (a) The relationship between PTPRT and CD4+ T cell infiltration. (b) The relationship between PTPRT and CD8+ T cell infiltration. (c) The relationship between PTPRT and Treg cell infiltration. (d) The relationship between PTPRT and B cell infiltration. (e) The relationship between PTPRT and Monocyte cell infiltration. (f) The relationship between PTPRT and macrophage M0 cell infiltration. (g) The relationship between PTPRT and macrophage M1 cell infiltration. (h) The relationship between PTPRT and macrophage M2 cell infiltration. (i) The relationship between PTPRT and Neutrophil cell infiltration. (j) The relationship between PTPRT and dendritic cell infiltration. (k) The relationship between PTPRT and NK cell infiltration. (l) The relationship between PTPRT and Hematopoietic cell infiltration.

PTPRT was positively associated with CD8+ T cell (*r* = 0.18), CD4+ T cell (*r* = 0.25), Neutrophil cell (*r* = 0.44), stem cell (*r* = 0.47), and M2 macrophage cell (*r* = 0.30) infiltration but negatively associated with B cell (*r* = −0.31), DC cell (*r* = −0.33), NK cell (*r* = −0.18), monocyte cell (*r* = −022), M0 macrophage cell (*r* = −0.21), and M1 macrophage cell (*r* = −0.35) (Figure 8). High PTPRT independently predicts better outcome (HR = 0.91, 95% CI 0.86 0.97, *p* = 0.002) in breast cancer corrected for patient age, stage, and TIICs (Table 1).

## 4. Discussion

PTPRT is an antioncogene and plays important roles in various cancers, including colorectal cancer [2], hepatocellular carcinoma [3], prostate cancer [4], lung squamous cell carcinoma [5], and glioma [7]. Several studies showed overexpressed PTPRT might inhibit tumor cell growth acting as a putative tumor suppressor in cancer cell culture [2–5, 7, 8]. Animal studies showed PTPRT knockout increases the size of mouse colon tumors in the Apcmin+/- genetic background, suggesting that inactivation of PTPRT promotes tumor progression [14] Our study analyzed the role of PTPRT in breast cancer, and we found the PTPRT mRNA level could be biomarkers for different stages, age groups, molecular types, and grades for breast cancer, as well as prognostic biomarkers for breast cancer. Based on our analysis, it is obvious that a larger tumor was associated with a lower PTPRT expression level. Meanwhile, breast tumor with high PTPRT was associated with low proliferation rate (measured by Ki67) and high apoptotic rates (measured by BCL-2). All these data suggest PTPRT might inhibit tumor growth in breast cancer as a tumor suppressor. The signal transducer and activator of transcription 3 (STAT3) protein is a major transcription factor involved in many cellular processes, such as cell growth and proliferation, differentiation, migration, and cell death or cell apoptosis [15]. Plenty of evidence suggested PTPRT might negatively regulate STAT3 activation by dephosphorylation of the tyrosine residue [15–18]. STAT3 may be activated by loss-of-function of negative regulators of STAT3, including by promoter hypermethylation of PTPRT [17]. This was confirmed in breast cancer, and PTPRT was negatively associated with STAT3, while the promoter methylation level of PTPRT was positively associated with STAT3 based on TCGA data.

PTPRT might predict the effectiveness of primary resistance biomarkers for taxane, anthracycline, and ixabepilone, which all displayed better effectiveness in breast cancer disease control [19–21], but not be acquired resistance biomarkers. Taxane were potent cytotoxic microtubule-stabilizing agents, and they exert their action through induction of apoptosis through phosphorylation of bcl-2 and inhibition of cell proliferation [22], as well as selectively disrupting the microtubule dynamics, inducing mitotic arrest that leads to cell death [23]. Anthracyclines, which belong to cell cycle nonspecific agents, are a class of potent and widely used cytotoxic drugs, derived from antibiotics that inhibit DNA and RNA synthesis by intercalating between base pairs of the DNA/RNA strand [24]. Ixabepilone bind to the *β*-tubulin subunit of the *α*, *β* dimer of microtubules, inducing microtubule polymerization, stabilization, and formation of abnormal mitotic spindles, which in turn cause G2/M arrest and apoptosis [25, 26]. The cell signaling pathways regulated by PTPRT largely remain to be elucidated. Based on our GO and KEGG analysis, we could find PTPRT might be associated with the cell cycle and microtubule-based process. It was reported that microtubules are cytoskeletal structures that play a pivotal role in cell division, locomotion, and intracellular transport [27]. During mitosis, microtubules, which consist of *α*- and *β*-tubulin, represent a major structural component of the spindle apparatus, which is required for the separation of sister chromatids [28]. Our analysis indicated that PTPRT was significantly associated with several genes that were involved in microtubule motor activity. This might explain why PTPRT could be a primary resistance biomarker for taxane, anthracycline, and ixabepilone.

Acquired drug resistance to chemotherapy and targeted therapy treatment is unavoidable, creating a clinically challenging problem, which represents a major challenge in for various types of cancers [29, 30]. Acquired resistance develops after a significant initial response over the course of several months [31]. Hammerlindl et al. [31] proposed that treatment will initially facilitate cellular reprogramming towards the slow-cycling drug-tolerant phenotype and continuous drug exposure will eventually lead to reactivation of transcriptional activity and regain of proliferative capacity. These will further stabilize their drug-tolerant transcriptional profile to become permanent drug resistant. According to their theory, PTPRT stays stable during the acquired resistance process, which means the expression of PTPRT did not change during the long drug exposure. So PTPRT might be a good primary resistance biomarker for taxane, anthracycline, and ixabepilone without affecting by the drugs.

Based on our data, although PTPRT was coexpressed with ESR1 and ERBB2, the status of ESR1 and ERBB2 did not affect the expression of PTPRT. Whether PTPRT affects the expression of ESR1 and ERBB2 was unclear. Based on our study, higher PTPRT was associated with longer survival in different molecular types based on KMplot data, and this was confirmed after adjusting several clinical factors based on TCGA data.

Our study comprehensively analyzed the role of PTPRT in breast cancer. In our study, not only TCGA but also GEO data were included to explore the role of PTPRT in breast cancer. We found PTPRT might predict the effectiveness of taxane, anthracycline, ixabepilone, and the prognostic values. We confirmed that PTPRT might inhibit tumor growth in breast cancer, which might be due to microtubule dynamics. However, our study still has its own limitations: first, all of our analyses were based on RNA sequence data. Our study was based on RNA sequence, whether q-pcr or IHC results still have the predictive values for the effectiveness of taxane, anthracycline, and ixabepilone and prognosis or not. Second, population heterogeneity might exist in this study across different datasets, although we used GEO datasets to validate the results in TCGA data. Third, PTPRT might be an inhibitor of tumor growth via disrupting the microtubule dynamics and cell cycle in breast cancer. This lacked in vivo and in vitro experiments to validate our study results. Future studies are needed about how PTPRT affects the drug effectiveness and breast cancer prognosis, as well as microtubule dynamics and cell cycle.

## 5. Conclusion

PTPRT expression was not affected by ER or HER2 expression, but PTPRT could distinguish Luminal A and TNBC, HER2+ breast cancer. PTPRT could be used as biomarkers to predict taxane, anthracycline, and ixabepilone effectiveness and prognosis for breast cancer patients. PTPRT might be an inhibitor of tumor growth via disrupting the microtubule dynamics and cell cycle in breast cancer.

## Figures and Tables

**Table 1:** Multivariable Cox proportional hazard model for PTPRT in breast cancer.

	Coef	HR	95%CI_lower	95%CI_upper	*p* value
Age	0.034	1.034	1.019	1.05	<0.0001
Gender: male	0.154	1.167	0.159	8.561	0.879
Race: Black	-0.4	0.67	0.196	2.297	0.524
Race: White	-0.58	0.56	0.173	1.812	0.333
Stage 2	0.479	1.615	0.848	3.075	0.145
Stage 3	1.307	3.693	1.903	7.167	<0.0001
Stage 4	2.58	13.201	5.845	29.817	<0.0001
Purity	0.264	1.302	0.448	3.788	0.628
B_cell	-1.393	0.248	0.002	28.446	0.565
CD8_Tcell	-1.735	0.176	0.012	2.571	0.204
CD4_Tcell	1.062	2.891	0.053	156.739	0.602
Macrophage	3.336	28.094	1.591	496.112	0.023
Neutrophil	1.629	5.1	0.018	1441.713	0.572
Dendritic	-0.735	0.48	0.055	4.2	0.507
PTPRT	-0.092	0.912	0.86	0.968	0.002

## Data Availability

The data used to support the findings of this study are included within the article.
